# Distinct modulation of inactivation by a residue in the pore domain of voltage-gated Na^+^ channels: mechanistic insights from recent crystal structures

**DOI:** 10.1038/s41598-017-18919-1

**Published:** 2018-01-12

**Authors:** Rene Cervenka, Peter Lukacs, Vaibhavkumar S. Gawali, Song Ke, Xaver Koenig, Lena Rubi, Touran Zarrabi, Karlheinz Hilber, Walter Sandtner, Anna Stary-Weinzinger, Hannes Todt

**Affiliations:** 1 Center for Physiology and Pharmacology, Department of Neurophysiology and Neuropharmacology, Medical University of Vienna, Waehringerstrasse 13a, 1090 Vienna, Austria; 2 Department of Pharmacology and Toxicology, University of Vienna, Althanstrasse 14, UZA 2, A-1090 Vienna, Austria; 3 Center for Physiology and Pharmacology, Medical University of Vienna, Waehringerstrasse 13a, 1090 Vienna, Austria

## Abstract

Inactivation of voltage-gated Na^+^ channels (VGSC) is essential for the regulation of cellular excitability. The molecular rearrangement underlying inactivation is thought to involve the intracellular linker between domains III and IV serving as inactivation lid, the receptor for the lid (domain III S4-S5 linker) and the pore-lining S6 segements. To better understand the role of the domain IV S6 segment in inactivation we performed a cysteine scanning mutagenesis of this region in rNav 1.4 channels and screened the constructs for perturbations in the voltage-dependence of steady state inactivation. This screen was performed in the background of wild-type channels and in channels carrying the mutation K1237E, which profoundly alters both permeation and gating-properties. Of all tested constructs the mutation I1581C was unique in that the mutation-induced gating changes were strongly influenced by the mutational background. This suggests that I1581 is involved in specific short-range interactions during inactivation. In recently published crystal structures VGSCs the respective amino acids homologous to I1581 appear to control a bend of the S6 segment which is critical to the gating process. Furthermore, I1581 may be involved in the transmission of the movement of the DIII voltage-sensor to the domain IV S6 segment.

## Introduction

Voltage-gated Na^+^ channels (VGSC) permit rapid transmission of depolarizing impulses throughout cells and cell networks, which is essential for the function of skeletal muscle, heart and the nervous system. Upon a strong depolarization VGSCs first open and then enter into several non-conducting inactivated states. New openings are only possible if inactivation is removed by a voltage-dependent recovery process. The molecular basis of fast inactivation is incompletely understood. A stretch of amino acids in the intracellular linker connecting domains (D) III and IV has been shown to occlude the inner opening of the permeation pathway, thereby preventing conduction^1,2^. However, it is unclear which molecular events following channel opening render the channel permissive to the binding of the inactivation particle. The internal part of the conducting pore is formed mainly by the S6 segments (S) of the four domains. Thus, the formation of a pre-inactivated pore conformation is most likely due to some molecular motion of one or several S6 segments. Indeed, mutations in the S6 segments have been shown to affect inactivation^3–7^. Notably, the S6 segment of domain IV (DIV-S6) has been suggested to play a dominant role in the process of inactivation^5,8^.

We investigated gating perturbations by serial cysteine replacements for DIV-S6 residues 1575-91 in the rNav1.4 channel. Such gating perturbations may be produced by long-range interactions resulting in global changes in the protein structure, or by short-range interaction between specific amino acids. In order to distinguish between these possibilites, we performed the serial cysteine replacements of DIV-S6 residues in two different backgrounds: in wild type channels, and in channels carrying the mutation K1237E, which per se is likely to give rise to global structural changes of the DIV S6 segment^9^ (see below). We reasoned that mutation-induced gating perturbations due to changes in long-range interactions would be similar in both backgrounds, whereas gating perturbations due to disruption of short-range interactions would highly depend on the mutational background.

We found the mutation I1581C to be exceptionally sensitive to the mutational background with regard to changes in the voltage at which half-maximum fast inactivation occurred (V_1/2_). This suggests a specific role of this position in the control of fast inactivation. Recently published crystal structures of several prokaryotic and one eukaryotic voltage-gated Na channel suggest that the site homologous to rNav1.4 1581 may be involved in the control of the position and the conformation of the S6 segments, thereby modulating activation and inactivation.

## Results

In order to screen DIV-S6 for gating-sensitive positions we applied a “two hit” strategy. First, we tested the effect of single serial replacements of amino acids by cysteine. Then we attempted to acquire additional information by testing the same constructs in the background of a mutation that severely impacts both permeation and gating.

### First hit: Cysteine replacements in DIV-S6 give rise to shifts of the voltage-dependence of fast inactivation

**Figure 1:**
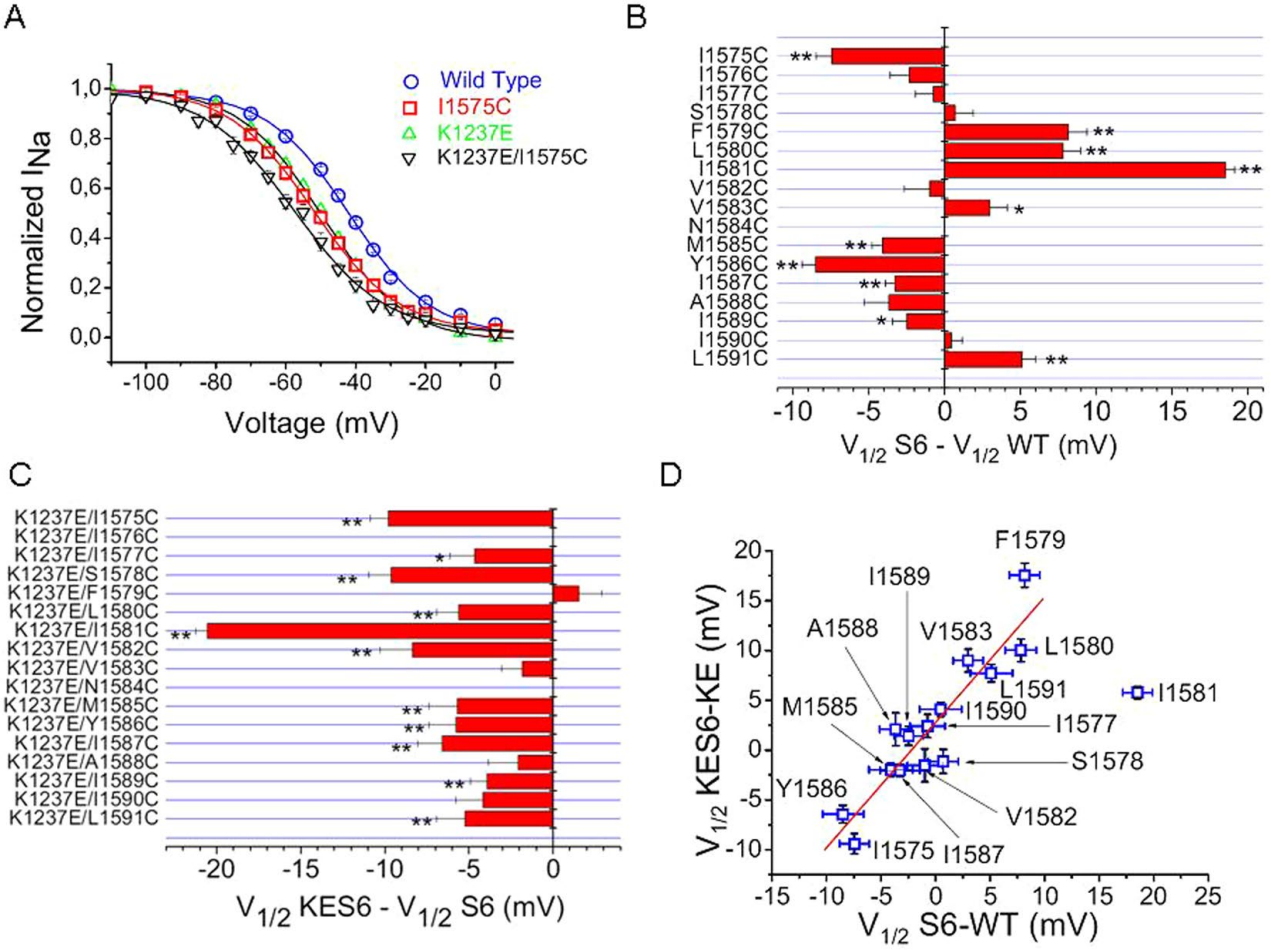
Gating perturbations by serial cysteine mutagenesis of DIV-S6. (**A**) Mutation-induced changes in the voltage-dependence of fast inactivation at site 1575 in DIV-S6. The normalized currents were fit by a Boltzman equation (Eq. 1). The estimated values for V_1/2_ were −43.7 ± 0.5 mV and −51.8 ± 1.1 mV for wild type and I1575C channels, respectively (P < 0.01. n = 7–8). The respective values for the slope factors were 10.1 ± 0.3 mV, and 11.5 ± 0.3 mV for wild type and I1575 channels, respectively (P = n.s.). The mutation in the selectivity filter of DIII. K1237E also caused a hyperpolarizing shift in the V_1/2_ relative to wild type (−51.1 ± 1.2 mV, n = 5, P < 0.001 vs. wild type). The combination of I1575 with K1237E resulted in a further negative shift of V_1/2_ (−59.6 ± 1.4 mV, n = 5, P < 0.001 vs. wild type, P = 0.01 vs. K1237E). The slope factors were unaffected by K1237E (10.8 ± 0.5 mV) and K1237E/I1575C (11.3 ± 0.4 mV). (**B**) Effect of cysteine-scanning mutagenesis on the V_1/2_ of fast inactivation. Bars indicate the mutation-induced change in V_1/2_ with respect to wild type (n = 5–9, *P < 0.05, **P < 0.01). (**C**) K1237E gives rise to a hyperpolarizing shift in V_1/2_. Shown are the shifts in V_1/2_ produced by adding the mutation K1237E to the indicated mutations in DIV-S6 (n = 5–9; *P < 0.05, **P < 0.01). The constructs K1237E/I1576C and K1237E/N1584C did not express current. (**D**) Effect of background on gating-perturbation by cysteine-scanning mutagenesis in DIV-S6. Shown are the shifts in V_1/2_ produced by cysteine substitutions in DIV-S6 in the background of wild type (abscissa) and in the background of the mutation K1237E (ordinate). There is a clear correlation between the examined gating perturbations with the notable exception of position 1581, which is an obvious outlier and. therefore. was omitted from the regression analysis (R = 0.94, P < 0.0001).

We tested the effect of mutations of residues downstream to position 1575, because this site is most probably located at the level of the selectivity filter and, thus, constitutes the upper limit of the internal cavity^10^. Figure 1A shows an example of the steady state inactivation curve for wild type channels and for channels carrying the mutation I1575C, i.e. the site in DIV-S6 that is located most external of all investigated positions. This mutation gave rise to a significant ~8 mV hyperpolarizing shift of the V_1/2_ without changing the slope factor. Figure 1B depicts the changes in V_1/2_ by serial mutagenesis with respect to wild type as a function of amino acid position in DIV-S6. Mutant I1584 did not express ionic currents and was not studied. Cysteine replacements gave rise to V_1/2_ shift both to depolarized and to hyperpolarized potentials. The amount of V_1/2_ shift produced by the mutations was small, with the notable exception of the mutation I1581C, which gave rise to a ~20 mV depolarizing shift of V_1/2_.

### Second hit: a mutation in the selectivity filter produces profound alterations in ionic selectivity and gating

K1237 is considered an essential part of the selectivity filter as mutations at this site have been shown to reduce the selectivity for Na^+^ ions and even allows for permeation of large organic cations^11–15^. Previously, we reported that this mutation gives rise to a long-lived inactivated state termed “ultra-slow inactivation”^9,16,17^. Here we investigated whether “shorter” forms of inactivation are also modified by K1237E.

**Figure 2:**
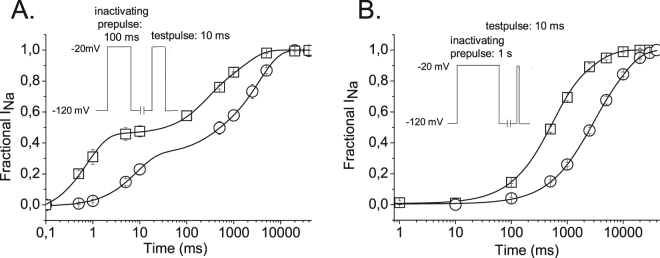
Time course of recovery from inactivation in wild type and K1237E channels. (**A**) Short prepulse duration: Ooytes were subjected to a 100 ms depolarizing prepulse to −20 mV. The normalized inward currents elicited by the test pulses were fit with the sum of three exponentials (Eq. 3). A_1_, A_2_ and A_3_ were 0.50 ± 0.02. 0.25 ± 0.06, and 0.25 ± 0.06 for wild type and 0.33 ± 0.03, 0.11 ± 0.03 and 0.56 ± 0.01 for K1237E, respectively (P < 0.01, n = 3). τ_1_, τ_2_ and τ_3_ were 0.8 ± 0.05 ms, 231.8 ± 62.2 ms, 1667.8 ± 512.2 ms for wild type and 8.3 ± 1.4 ms, 295.8 ± 100.4 ms and 3443.8 ± 114.7 ms for K1237E, respectively (P < 0.01, n = 3). (**B**) Long prepulse duration: clamp protocols was as in described in A. with the exception that the prepulse duration was 1 s. The data points were fit with the sum of two exponentials (Eq. 2). A_1_ and A_2_ were 0.70 ± 0.05 and 0.30 ± 0.05 for wild type and 0.45 ± 0.05 and 0.55 ± 0.05 for K1237E, respectively (P < 0.01, n = 6–9). τ_1_ and τ_2_ were 486.5 ± 41.9 ms and 2588.1 ± 403.9 ms for wild type and 1804.1 ± 181.6 ms and 8140.6 ± 566.7 ms for K1237E, respectively.

When expressed in *Xenopus laevis* oocytes rNav 1.4 channels recover from inactivation with up to three time constants, reflecting recovery from fast inactivation, and from at least two slow inactivated states^18^. As shown in Fig. 2 this holds true for both wild type and K1237E channels subjected to a conditioning prepulse of 100 ms. Increasing the duration of the prepulse to 1 s gave rise to exclusive recovery from two slow inactivated states. With both protocols the mutation K1237E substantially slowed the time course of recovery both by increasing the time constants of recovery (Fig. 2A) and by increasing the fraction of channels recovering from slow inactivation (as reflected by the increased values for A_2_ and A_3_; Fig. 2A,B). The fact that this construct produces substantial alterations of both permeation properties and gating behavior suggests that K1237E results in a broad conformational change of the channel protein. In the following experiments we exploit this property to uncover gating-sensitive mutations in DIV-S6 by double mutagenesis.

### Second hit: Effect of combination of mutations in DIV-S6 with K1237E

Figure 1A shows that K1237E alone shifts the V_1/2_ of fast inactivation by ~−6 mV. A similar negative shift in V_1/2_ is observed if the mutation K1237E is added to the mutation I1575C in DIV-S6. In this case the effect of K1237E was independent of the change resulting from the mutation in DIV-S6. Figure 1C shows that this is not the case for all mutations in DIV-S6. Site 1581 was most sensitive to the amino acid replacement in the selectivity filter, shifting V_1/2_ by more than −20 mV, i.e. more than twofold greater than any other site. Thus, the mutation K1237E had nonuniform effects on the gating perturbations produced by serial S6 cysteine mutagenesis. We plotted the shifts in V_1/2_ by mutations in DIV-S6 relative to K1237E as a function of the respective changes relative to wild type. Figure 1D demonstrates that, with one exception, the kinetic effect of mutations in DIV-S6 in the background of wild type channels were significantly correlated with the effects of the same mutations in the background of K1237E. Site 1581 is a notable exception to this pattern. While the single cysteine replacement shifted the V_1/2_ dramatically to more positive potentials, the addition of K1237E gave rise to an almost equally dramatic shift to more hyperpolarized potentials. Hence, the position 1581 is exceptionally sensitive to the investigated gating perturbations.

### Are the shifts in SSI produced by the mutations in DIV-S6 a result of shifts in activation?

**Figure 3:**
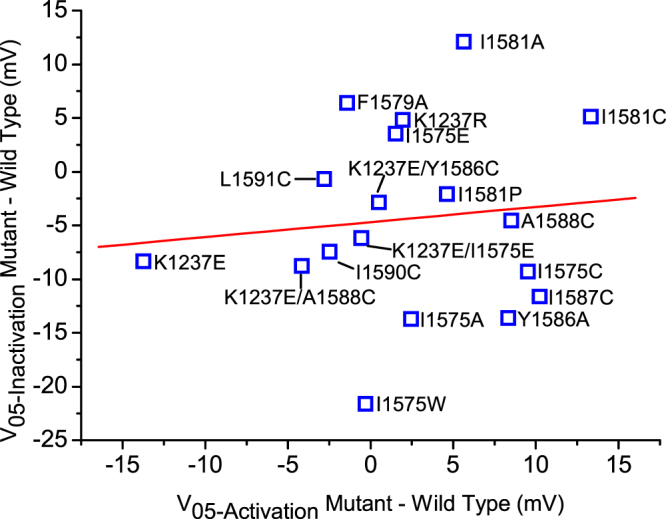
The observed shifts in V_1/2_ of inactivation are independent from shifts in activation. Each data point is labelled with the respective mutation. The voltage-dependence of activation was determined as described in Methods and the normalized currents were fit with Eq. 4 in order to derive the V_1/2_ for activation (R = −0.22, P = 0.54).

Fast inactivation derives its voltage-dependency in part from voltage-dependency of activation^19^. Thus we wondered whether the mutation-induced shifts in the voltage-dependence of steady-state fast inactivation could be secondary to a shift in the voltage dependence of activation. To investigate this possibility we studied the constructs by means of whole-cell the patch-clamp technique in heterologously transfected mammalian TsA201 cells, which allows for good voltage-control of the fast activation process. Some of the constructs carrying the mutation K1237E either did not express or produced only small currents in mammalian cells. Unfortunately, not all double mutants could be expressed in TsA201 cells. Figure 3 shows plot of the mutation-induced shift in inactivation as a function of the respective shift in activation in those constructs that expressed sufficient current. The changes in the voltage-dependence of activation and fast inactivation produced were not correlated with each other. Thus, the reported shifts in inactivation are probably independent of changes in the voltage-dependence of activation.

### K1237 does not directly interact with DIV-S6

**Figure 4:**
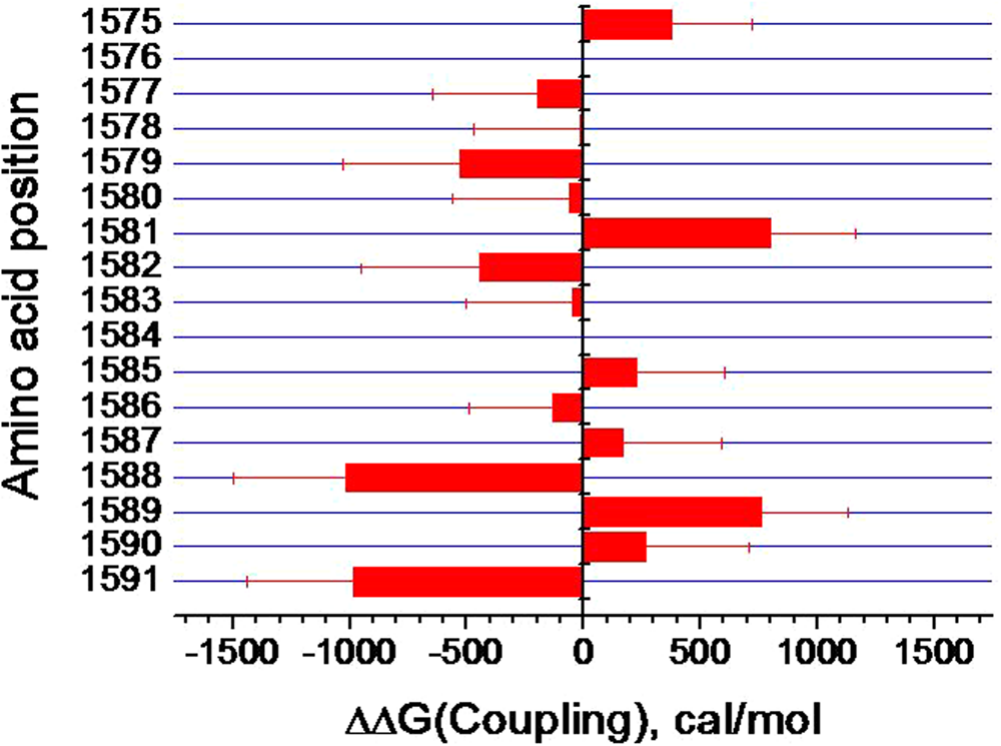
The selectivity filter does not couple directly to DIV-S6 during inactivation. Histogram of the coupling energies between site 1237 in the selectivity filter and the indicated positions in DIV-S6. Coupling energies were calculated from the values of V_1/2_ and k which were derived from the fits of a Boltzmann equation (Eq. 1) to the normalized data of steady state inactivation. as described in “Methods” and Fig. 1.

The profound effects of the mutation K1237E on the V_1/2_ of inactivation in the tested constructs (Fig. 1C) raises the question whether some of the gating perturbations resulted from a direct interaction between DIV-S6 and the selectivity filter. Thermodynamic mutant cycle analysis has previously been performed to identify direct molecular interactions during inactivation^20–23^. We applied this method to the mutant cycles consisting of wild type, K1237E, single cysteine replacements in DIV-S6 and combination of the latter two constructs. Figure 4 shows the coupling energies of the mutant cycle pairs at the tested sites in DIV-S6. However, none of the coupling energies approaches values of >1 kcal/mol which is considered to indicate direct molecular coupling^24,25^.

### Hydrophobic interactions at site 1581 contribute to modulation of gating

**Figure 5:**
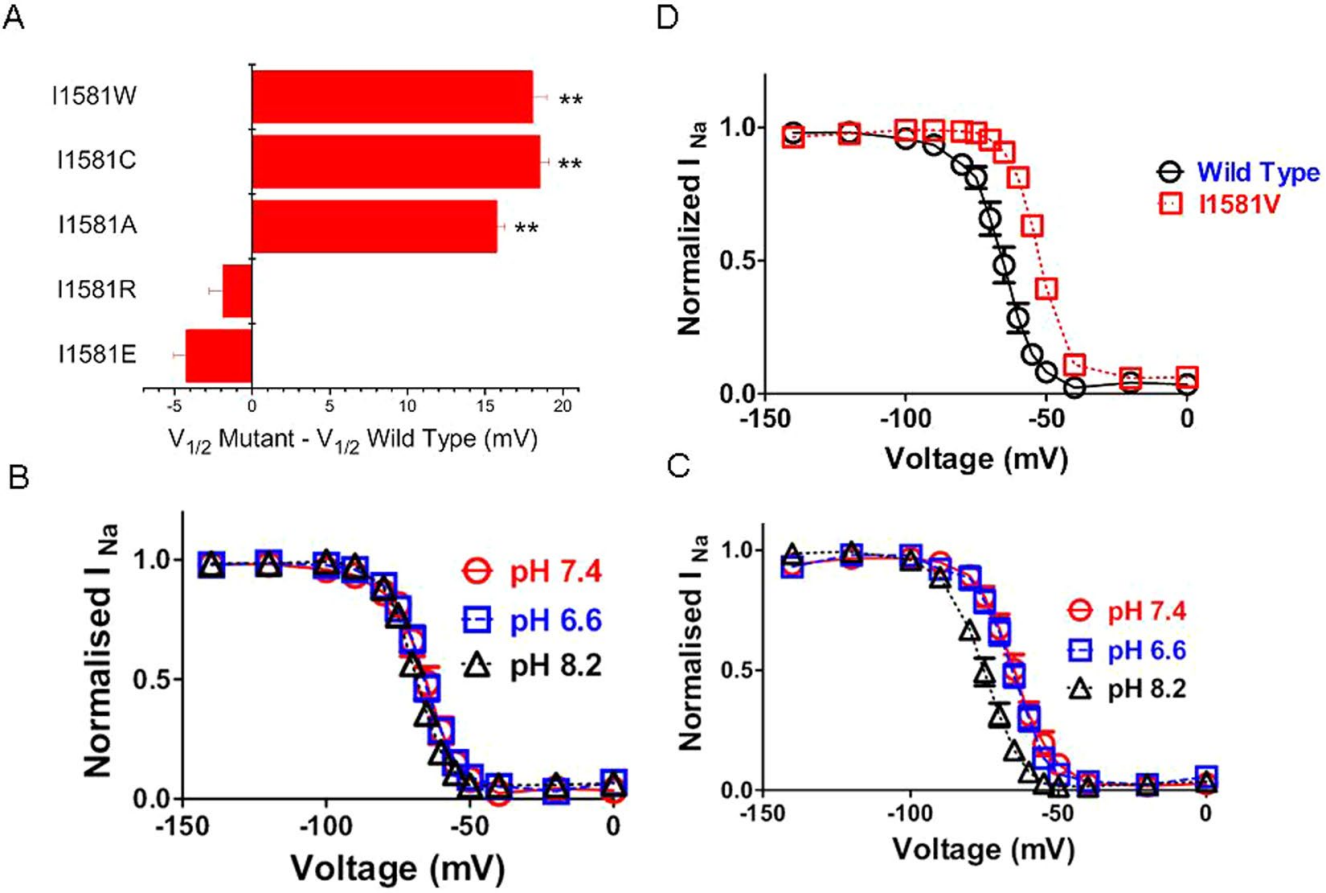
Gating perturbations by amino acid substitutions of I1581. (**A**) Hydrophobic substitutions at site 1581 give rise to large shifts in V_1/2_ of inactivation. I1581W significantly shifted the V_1/2_ to more positive values compared to wild type (**P < 0.01. n = 5–9). For comparison the data for I1581C are reproduced from Fig. 1B. (**B**,**C**) A histidine engineered to site 1581 allows for modulation of inactivation by pH. V_1/2_ values were for wild type −66.5 ± 1.2 mV, −71.1 ± 2.5 mV, −69.1 ± 1.0 mV for pH values of 6.6, 7.4 and 8.2, respectively (n = 7–9). V_1/2_ values were for I1581H −66.4 ± 1.7 mV, −65.8 ± 1.5 mV, and −75.3 ± 1.3 mV for pH values of 6.6, 7.4, and 8.2, respectively (n = 4–6). The only significant difference was with I1581 for V_1/2_ at pH 7.4 and pH 8.8. (**D**) The mutation I1581V recapitulates isoform differences in gating. I1581V was transfected into mammalian tsA201 cells and examined using the patch-clamp technique. The holding potential was -140 mV. The values for V_1/2_ were −66.2 ± 0.6 mV for wild type and -53.8 ± 0.6 mV for I1581V (n = 5–9. P < 0.05).

In order to gain insight into the molecular mechanism of the gating sensitivity at site 1581 we systematically replaced I1581 by amino acids of different size and hydrophilicity. As shown in Fig. 5A the constructs carrying hydrophobic substitutions produced a substantial depolarizing shift in the voltage-dependence of inactivation, whereas constructs in which isoleucine was substituted by the hydrophilic positively and negatively charged amino acids showed only minor hyperpolarizing shifts. Histidine is a residue whose degree of protonation can be altered by pH. The pKa of histidine in solution is 6.0, however in protein environment pKa values between 5.0 and 8.0 have been reported^26^. As shown in Fig. 5B,C changing the pH of the external solution between 6.6 and 8.2 had no effect on the V_1/2_ of fast inactivation in wild type channels. Interestingly, this insensitivity to pH changes of wild type channels appears to be a special feature of Nav1.4, whereas the kinetic behavior of Nav1.2 and Nav1.5 channels is modulated by external pH^27^. Substitution of I1581 with histidine had no effect in acidic environment but substantially shifted the V_1/2_ of inactivation at higher pH values at which histidine is expected to be less hydrophilic. Substitution of isoleucine by a bulky tryptophane had a similar effect as substitution by the small alanine, suggesting that the size of the side chain does not play a major role in the modulation of inactivation.

### Recently published crystal structures VGSCs suggests a pivotal role of a site homologous to 1581 in activation gating

**Figure 6:**
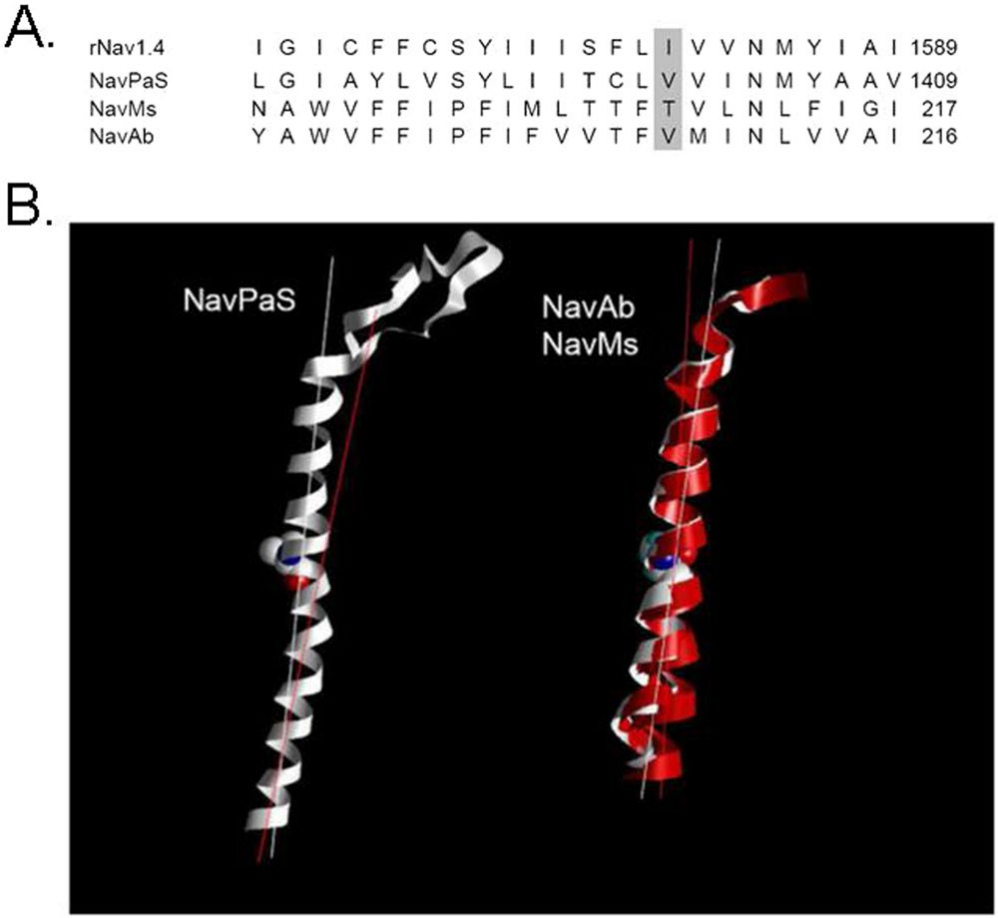
Potential gating mechanism of I1581 and biological significance. (**A**) Site 1581 is homologous to a pivot point for activation in NavMs. Sequence alignment between DIV-S6 of rNav 1.4., the recently crystallized eukaryotic NavPaS^33^, and the prokaryotic VGSC structures of NavMs^30^ and of NavAb^28^. The alignment is based upon figure S1 in Shen *et al*.^33^, Fig. 1 in McCusker *et al*.^30^, Supplementary Figure 7 in Payandeh *et al*.^28^. I1581 aligns with V1401 of NavPaS, with T209 of NavMs and with V208 of NavAb (shaded area). (**B**) Crystal structures of DIV S6 of NavPaS (left, PDB#5x0 M) and an alignment of the pre-open NavAb^28^ (PDB#3RVZ)) and the presumably open state of NavMs^30^ (right, (PDB#4F4)). In all stuctures the S6 segment has a bend starting with the amino acids homologous to rNav1.4 I1581 (see A., amino acid side chain indicated). For better identification of the bend the long axis-centers of the proximal and distal parts of the S6 segments are indicated by lines. Right panel: The structures of NavAb of NavMs are aligned with reference to the selectivity filter. The NavMs structure does not contain voltage-sensors, therefore no S4-S5 linker is available. All four S6 segments of the tetrameric NavMs are shown (red ribbons) These S6 segments are clearly displaced with respect to the pre-open NavAb structure (white ribbon), albeit to slightly different degrees. This displacement is generated by a rotation around the backbone angle of T209 (side chain indicated), which swings that helix away from the central pore opening up the bottom of the structure^30^.

Recently a number of crystal structures of VGSCs have been solved^28–34^. We first consider the structures of the prokaryotic VGSCs NavAb and NavMs^28–30^. The structure of NavMs is proposed to represent the open state of the channel. Comparison with the closed structure NavAb-I217C suggests that channel opening occurs by a rotation around the backbone angle of T209 in the middle of S6 helix. As a result S6 swings away from the central pore, opening up the bottom of the channel (Fig. 6B, right panel). Interestingly, this key residue T209 is homologous to I1581 in rNav1.4 (Fig. 6A). Recently the first structure of a eukaryotic VGSC has been published^33^. As shown in Fig. 6B the DIV S6 segment also contains a backbone angle close to V1401, which is homologous to T209 in NavMs and I1581 in rNav1.4. These structural data support a significant role of position I1581 in gating of VGSCs. It may be argued that the mentioned conformational change at site 209 in NavMs relates to channel activation whereas this study concerns the mechanism of channel inactivation. It has been suggested that channel opening occurs upon activation of all four voltage sensors, whereas activation of only two voltage-sensors in DIII and DIV accounts for closed state inactivation^35^.

### Possible interaction of position 1581 with the DIII S4-S5 linker

**Figure 7:**
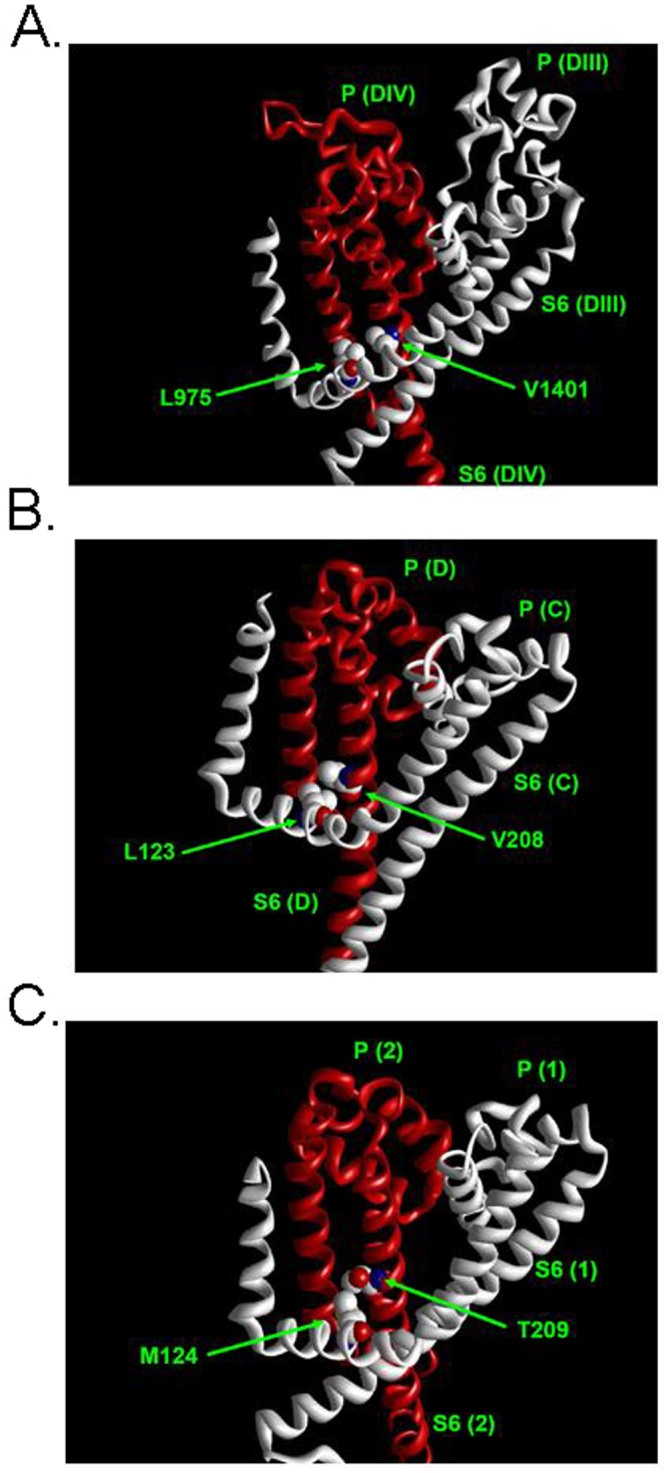
Spacial relationship between the S4-S5 linker of one domain/subunit and the S6 segment of an adjacent subunit/domain in recently published VGSC structures. The backbone of one subunit/domain is indicated by a ribbon of a single color. Shown are only side chains of the amino acids homologous to rNav1.4 L1146 (S4-S5 linker) and I1581 (DIV S6) which are in close proximity in all structures. “P” denotes P-loop. (**A**) NavPaS (eukaryotic)^33^} (PDB#5x0 M), DIII and DIV. (**B**) NavAb (prokaryotic, closed)^32^} (PDB#5XB2) chains **C** and **D**. (**C**) NavMs (prokaryotic, open)^31^} (PDB#5HVD). Unlike NavAb this structure has not been crystallized as tetramer thus two identical chains are shown (1, 2).

It has been suggested that the voltage-dependent movement of the voltage sensors is translated to a gating motion of the S6 segments via an interaction of these segments with the respective S4-S5 linker of a neigboring subunit^29,31,36^. Recently VGSC structures containing all four voltage-sensors become available^32,33^. Figure 7 shows the spacial relationship between the S4-S5 linker of one domain/subunit and the S6 segment of an adjacent subunit/domain in the structures NavPaS (eukaryotic)^33^, NavAb (prokaryotic, closed)^32^ and NavMs (prokaryotic, open)^31^. The amino acid I1581 in rNav1.4 is homologous to V1401 in NavPaS, V208 in NavAb, and T209 in NavMs. These residues are in close proximity to an amino acid of the S4-S5 linker of the neighboring subunit. This amino acid corresponds to L975, L123, and M124 in NavPas, NavAb, and NavMs, respectively. These residues are homologous to L1146 in rNav1.4. Hence, site 1581 may be involved in the coupling of the movement of the voltage-sensor in DIII to the DIV S6 segment.

### Insights from the recently published structure of EeNav1.4

**Figure 8:**
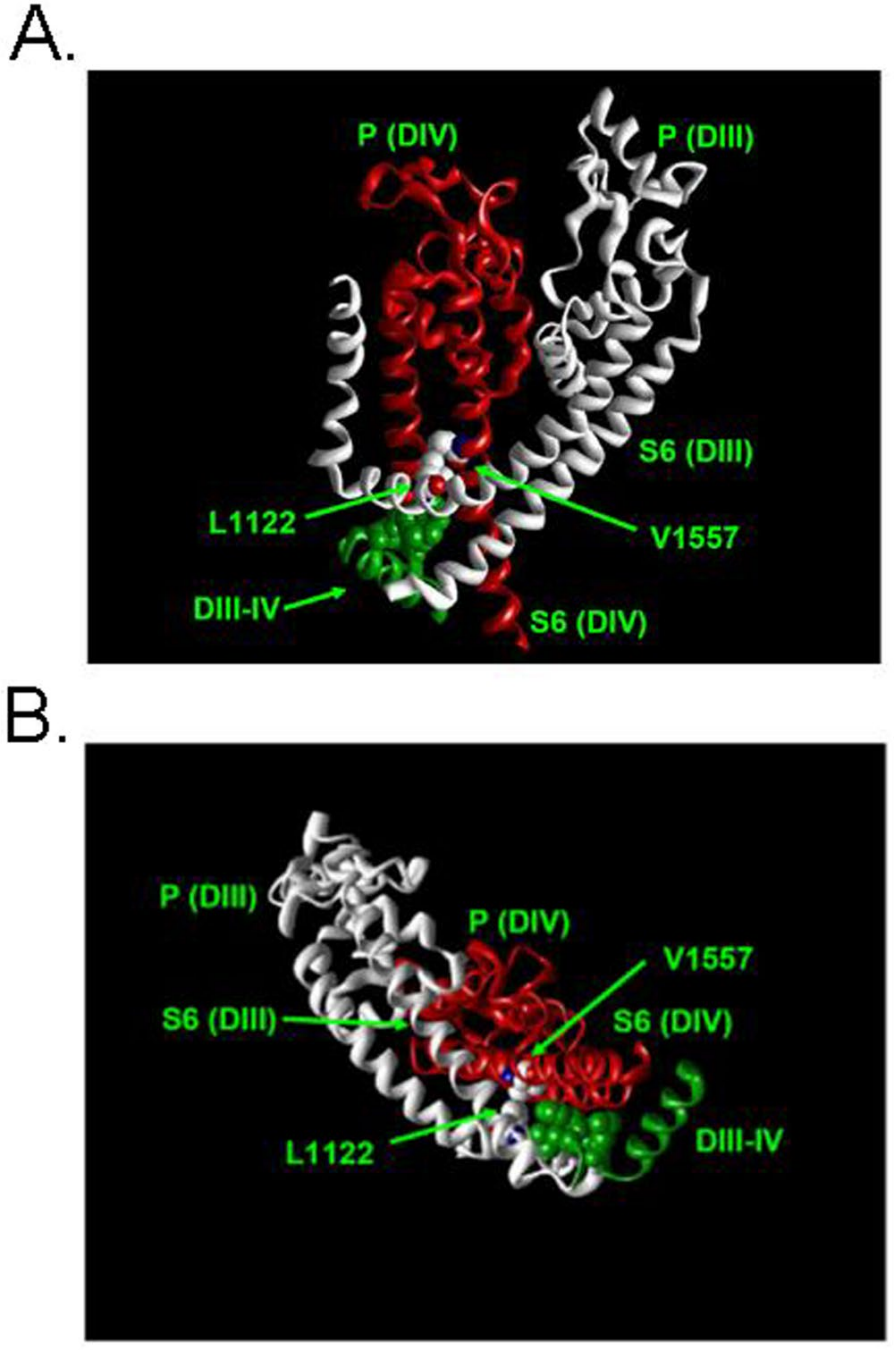
Spacial relationship of the inactivation gate (“IFM” motif) and the amino acids homologous to rNav I1581 and L1146 in the structure of EeNav1.4^34^ (PDB#5xsy). Shown are the backbones of S5, P-loop (“P”) and S6 of DIII (white) and DIV (red), the S4-S5 linker of DIII (white), and the intracellular linker between DIII and DIV (green) containing the IFM motif (here LFM) represented as spheres. Also represented as spheres are the amino acids homologous to rNav1.4 I1581 and L1146, V1557 and L1122, respectively. (**A**) Side view as with the structures in Fig. 7. (**B**) View from the intracellular side. Obviously, residues V1557 and L1122 are in close proximity to the inactivation gate (LFM).

Recently, the structure of the VGSC from electric eel (EeNav1.4) has been solved at 4.0 Å resolution^34^. Unlike the previously considered structures EeNav1.4 contains an intact inactivation gate composed of the IFM motif and an ensuing helix located in the intracellular linker between DIII and IV^1,2^. Although the intracellular gate is open EeNav1.4 is considered to represent an inactivated state. Interestingly, the IFM motif (which is LFM in EeNav1.4) does not occlude the intracellular mouth of the channel but is plugged into the corner enclosed by the S4-S5 and S6 segments of DIII and DIV. The authors suggest that this arrangement gives rise to a conformational change of the S6 segements that closes the permeation pathway. As shown in Fig. 8 V1557, which is homologous to rNav1.4 I1581 is in close proximity to L1122 of the DIII S4-S5 linker (L1146 in rNav1.4), confirming the previously mentioned arrangement in NavPaS, NavAb and NavMs (Fig. 7). Both amino acids are in close proximity to the bound IFM motif. Perhaps I1581 is involved in a hydrophobic interaction with the IFM motif thereby modulating fast inactivation.

### Amino acid at position 1581 varies between isoforms

The voltage-dependence of fast inactivation has been shown to vary substantially between isoforms of VGSCs^37^. An alignment of DIV-S6 reveals that within mammalian species there is a high degree of conservation among species of the amino acid homologous to site 1581 (Fig. S1). However, there is substantial variation at this position between isoforms. A valine is found at this position in rNav1.2, and the V_1/2_ of inactivation has been reported to be shifted positively in this isoform when compared to Nav1.4^37^. Given the observed substantial shifts by hydrophobic replacements at site 1581 (Fig. 5A) we wondered whether a valine engineered to this site would produce a similar shift, and, thus recapitulate the phenotype of Nav. 1.2.

As shown in Fig. 5D I1581V substantially shifts the V_1/2_ of fast inactivation to more positive values, consistent with a potential physiologic role of this site in the fine-tuning of isoform differences in inactivation gating.

### Molecular dynamics simulations suggest an interaction between site 1581 and the S4-S5 linker

The recently published crystal structure of the bacterial NavAb in potentially inactivated states suggested amino acid N211 of the S6 segment to interact with S4-S5 linker of an adjacent domain during inactivation^29^. N211 is homologous to N1584 in rNav1.4. In our hands the mutation N1584C did not express ionic current which may reflect the pivotal role of this position in inactivation gating. Amino acid V208, homologous to I1581 is positioned one helix turn “upward” (N-terminal) to N211.

To test for a potential molecular basis of a role of I1581 in inactivation gating we performed molecular dynamics simulations using a homology model of rNav 1.4 based on the published structure of NavAb-wild type chain A^29^. This structure is proposed to represent an inactivated state, produced by a two-fold symmetrical movement of the S6 segments towards the central pore axis. We chose to analyze NavAb-wild type chain A (inactivated) because N211 in this subunit forms a hydrogen bond with S4-S5 linker L123 from the adjacent subunit. We examined the non-bonded interaction energies of native I1581, and the substitution by valine with nearby (within 3.5 Å) located amino acids in the S6 segment, the S5 segment and the DIII S4-S5 linker (Supplementary Fig. 1). Interestingly the largest relative change in interaction energy produced by the mutation I1561V concerned the interaction between sites 1581 and L1164 in the DIII S4-S5 linker. As shown in Fig. 7 the amino acids homologous to rNav 1.4 I1581 and L1164 are in close proximity to each other in the crystal structures of NavPaS, NavAb, and NavMs.

## Discussion

To search for positions that are essential to the process of fast inactivation we performed serial cysteine scanning mutagenesis of DIV-S6 of Nav1.4. Although the effects of replacing DIV-S6 amino acids by alanines^38^ or tryptophanes^39^ have been reported, perturbations of fast inactivation gating by serial cysteine replacements in DIV-S6 have not been reported. Cysteine side chains are more reactive than those of alanines and may thus produce more severe gating perturbations. Our study is also new in that we examined the effect of serial cysteine replacements in DIV-S6 in the background of the mutation K1237E that most likely produced a global rearrangement of the protein.

Most cysteine mutants of DIV-S6 residues below the selectivity filter level resulted in some change in the voltage dependence of fast inactivation, confirming the prior evidence that DIV-S6 is involved in fast inactivation^40–47^. The largest effect by far was from mutation at site 1581, suggesting a special role for this site in the molecular process of fast inactivation (Fig. 1B). Yarov-Yarovoy *et al*. found only a small effect by alanine replacement at the homologous site in Nav1.2^38^. This may be because in Nav1.2 the naturally occurring residue is valine, instead of isoleucine (see later). In the background of the mutant K1237E changes were also produced in fast inactivation, but again the effect at site 1581 was uniquely large (Fig. 1C).

The mutation K1237E has been reported to shift the voltage-dependence of inactivation to hyperpolarized potentials^48^. This construct also gave rise to a hyperpolarizing shift in most S6 mutations, although the amount of shift varied between constructs (Fig. 1C). The especially large effect of the double mutant K1237E/I1581C could mean some special interaction between these sites. However, we were unable to demonstrate a direct interaction by mutant cycle analysis. This appears to hold true for all other investigated sites in DIV-S6, although direct interactions of the upper part of DIV-S6 with the selectivity filter may be critical for the generation of slower inactivated states^10^. Alternatively, K1237 may allosterically modulate the movement of DIV-S6. Allosteric modulation of fast inactivation by a residue in the P-loop (pore loop; DIV) has previously been shown for W1531^49^. This amino acid is predicted to be in close vicinity to K1237. Thus, when K1237 and W1531 are replaced by cysteines, disulfide bonds form between these sites^50^. Furthermore, the rate of this disulfide bond formation depends on inactivation gating, which supports the presence of allosteric link between the outer vestibule and the inactivation machinery. We recently reported that the side chain of W1531 most likely occupies a space between K1237 and I1575 in DIV-S6, such that replacement of W1531 opens a second ion conducting pore^51^. The idea that conformational changes in the selectivity filter region are coupled to molecular motions of the S6 segments is supported by the published crystal structures of presumably inactivated prokaryotic VGSCs NavAb and NavRh which exhibit conformational changes both in the selectivity filter region and in the intracellular part of the S6 segments when compared to the open structure of NavMs^29,52^. This led us to hypothetise that the mutation K1237E would offer a background in which short range interactions between amino acids are modified such that the cysteine scanning mutagenesis of DIV S6 results in kinetic changes substantially different from the same cysteine replacements in the wild-type background.

Apart from allosteric modulation, the alteration of charge in the selectivity filter region by K1237E may exert an electrostatic bias on voltage sensors, thereby shifting the voltage-dependency of inactivation rate constants. Such electrostatic interaction between the selectivity filter region and the voltage sensing apparatus has previously been suggested for binding of µ-conotoxin to the selectivity filter region^53^. However, a study of charge-altering mutations in the “second ring of charge” i.e. a ring of negatively charged amino acids, located 3 positions (4 in DII) C-terminal of the selectivity filter indicated that charge-altering mutations in this region are not associated with substantial changes in the voltage-dependency of activation arguing against an electrostatic interaction between the selectivity filter region and the voltage sensors^54^.

Finally, the well-known interaction between activation and inactivation could mean that the reported mutation-induced effects on inactivation are secondary to this activation effect, but no correlation was seen between the two effects (Fig. 3). Nevertheless, we cannot exclude that in individual constructs mutation-induced changes in inactivation may have resulted from alterations in activation.

Most likely, the selectivity filter mutant probably destabilizes the S6 scaffolding around the filter region, so that S6 movement for activation and inactivation is more easily achieved.

As shown in Fig. 1D the kinetic effect of cysteine replacements in DIV S6 was independent of the mutational background for all positions with the notable exception of site 1581, indicating that I1581 may be involved in specific short-range interactions with other amino acids thereby modulating the fast inactivation process. This idea is supported by structural data derived from several prokaryotic and eukaryotic VGSCs. Thus, the position 1581 may be involved in the formation of a bend in the S6 segment that could be critical to the rearrangement of the distal portion of the DIV S6 segment (Fig. 6). This bend is supposed to contol the opening of the distal activation gate by means of a lever mechanism^30,32^. With regard to fast inactivation, it is conceivable that by an analogous lever mechanism the position of the distal part of the S6 segments controls the formation of a high affinity binding site for the inactivation lid.

Apart from controlling the S6 bend, site 1581 may be involved in an interaction with the S4-S5 linker of DIII. This notion is supported by the recently published crystal structures of the eukaryotic NavPaS^33^ and the prokaryotic NavAb and NavMs^31,32^. All of these structures contain both voltage sensors and pore domains and allow insights into the coupling of voltage sensing to pore conformation. In these structures the amino acids homologous to rNav1.4 I1581 are in close proximity to a residue in the S4-S5 linker of the neighboring subunit/domain (Fig. 7). This residue is homologous L1146 in rNav1.4, which is conserved across all VGSC isoforms (see e.g. figure S1 in Shen *et al*.^33^). Interestingly, a mutation of this residue is associated with a human channelopathy resulting from altered inactivation properties of Nav1.2 channels (see below). Hence, the movement of the DIII voltage sensor could be transmitted to the DIV S6-segment via an interaction of L1146 and I1581. As shown in Fig. 7, the amino acids in the depicted crystal structures which are homologous to I1581 are facing away from the central pore. This position is supported by the fact that in inactivation-deficient rNav1.4, an introduced cytsteine at this site is inaccessible for chemical modification from the inside both in open, closed and inactivated states^55,56^. Furthermore, the notion that the DIII voltage sensor is involved in the mechanism of inactivation is supported by experimental data suggesting a special role of the voltage sensor of DIII in controlling fast inactivation^57,58^. However, this cannot be the only mechanism of coupling the voltage-sensor movement to the inactivation gate as also the DIV voltage-sensor is implicated in inactivation^43,44,47,57–60^. A model has been proposed in which the movement of voltage sensors of DIII and DIV set the stage for closed state inactivation^35^. Apart from a potential role of the S4-S5 linker in the transmission of DIII voltage sensor movement to the DIV S6 segment, this structure has been proposed to act as receptor for the fast inactivation particle^22^, again supporting a critical role of this region in fast inactivation. This notion is supported by the recently published eukaryotic VGSC structure EeNav1.4^34^ which is the first and as yet only structure containing a triplet of amino acids in the DIII-IV linker that is widely considered to compose the inactivation gate (“IFM motif”, LFM in EeNav1.4)^1,2^. In the potentially inactivated structure of EeNav1.4 this motif is bound to a pocket enclosed by the S4-S5 and S6 segments in DIII and IV^34^. As shown in Fig. 8 this motif is in close proximity to V1557, which is homologous to I1581 in rNav1.4. This suggests that the observed changes in inactivation properties by mutations at this site result from alterations of this binding pocket. The critical role of the S6 segments in the process of inactivation is also supported by the fact that each individual S6 segment undergoes rotation around its helical axis between the closed structure NavPaS and the inactivated EeNav1.4^34^. It is unclear how these complex molecular rearrangements produce a non-conducting inactivated state. The intracellular gate in EeNav1.4 is open but it it is suggested that the presence of the digitonin-like chemical may provide a strong counter force to preclude closing of the permeation pathway^34^.

It is striking that within a stretch of 15 amino acids that are completely conserved, the residue in site 1581 is unique in exhibiting isoform-dependent variation (Fig. 6C). Thus, in Nav1.4, 1.5, 1.6, 1.8, and 1.9 the position is occupied by an isoleucine, and in Nav1.1, 1.2, 1.3 and 1.7 by a valine (Fig. S1). The latter group of isoforms has been shown by maximum parsimony analysis to be most closely related and may have appeared late in evolution^61^. Isoleucine and valine are structurally and chemically similar. They are often found in homologous positions, substituting for each other in many proteins with little change in function^62^. When we mutated site 1581 to valine, instead of its native isoleucine, fast inactivation was shifted by +12 mV, almost as much as with the cysteine mutation (Fig. 5D). Consistent with this result, O’Leary reported that the V_1/2_ of inactivation of rNav 1.2 channels was shifted by about +10 mV compared with hNav1.4 channels^37^. Another similar residue to isoleucine is alanine, and we found that its substitution in rNav1.4 resulted in a shift of the voltage dependence of fast inactivation of about +16 mV, similar to that with the cysteine mutation (Fig. 5A). Obviously the structural requirements of site 1581 function during fast inactivation are rather stringent.

In an effort to determine the role of size of residue at site 1581, we substituted the large residue tryptophane, and produced the same depolarizing shift in fast inactivation as with cysteine, valine, and alanine. In contrast, replacement of isoleucine with glutamate or arginine failed to affect V_1/2_ of fast inactivation. The greatest effects were seen with hydrophobic residues, while hydrophilic residues had little effect. This interpretation is supported by the effect of pH change with the histidine mutation (Fig. 5C). In the presence of an acidic pH histidine should be more hydrophilic and it had little effect, but a shift of V_1/2_ was seen with high pH. Exact prediction of the structural role of site 1581 is not possible from these substitutions, but one plausible scenario is that a hydrophobic interaction between the residue at site 1581 (I or V) and L1146 of the DIII S4-S5 linker is involved in the process of fast inactivation. This idea is supported by molecular dynamics simulations indicating a substantial change in the energy of interaction between these sites by the mutation I1581V (Fig. S2). In summary, fast inactivation may result from a complex interaction involving the DIII-IV linker as well as parts of DIII and DIV. A recent study has provided experimental evidence for a time-dependent sequence of interactions between these molecular players^63^. Thus, following depolarization the DIII-IV linker first interacts with the DIV S4-S5 linker. Over ~100 ms, i.e. the time of depolarization used in this study, the DIII-IV linker interacts with the DIII-voltage sensor domain thereby stabilizing its activated conformation. The experimental as well as the modelling data of this study support such complex interaction between DIII, DIV and the DIII-IV linker.

Human channelopathies involving mutations at sites equivalent to rNav1.4 1581 and 1146. Given the potential role of rNav 1.4 sites 1581 and 1146 in control of fast inactivation it appears of interest whether mutations at these sites are involved in human channelopathies. To the best of our knowledge no channelopathies in any Na channel isoform has been reported at sites homologous to rNav1.4 1581. However, mutations in positions next to 1581 have been found to give rise to human disease. Thus the mutation Nav1.5 V1763M, located immediately C-terminal from the I1762, homologous to I1581 manifests as long QT syndrome^64^. This mutation gave rise to a persistend Na current and shifted the voltage-dependency of fast inactivation by 8 mV toward positive voltages. With regard to the potential interaction partner of I1581, L1146, located on the DIII S4-S5 linker, the mutation at the homologous site in Nav1.2, L1330F, has been detected in patients suffering from benign familial neonatal-infantile seizures. This mutation is associated with altered inactivation properties, although the exact biophysical changes appear to depend on the heterologous expression system^65,66^. These findings support a biological important role of the mentioned residues in controlling fast inactivation. Further studies are warranted to clearly define the nature of interaction between between the DIV S6 segment and the DIII S4-S5 linker.

## Methods

### Mutagenesis and Electrophysiology

Mutagenesis and electrophysiology were performed as reported^10,51^.

### Mutagenesis of rNa_V_1.4

A vector consisting of the rNa_V_1.4 coding sequence flanked by *Xenopus* globin 5′ and 3′ untranslated regions was provided as a gift by R. Moorman (University of Virginia, Charlottesville, VA, USA). This was used as the template for inserting oligonucleotide-directed point mutations by either four primer PCR and subsequent subcloning into the template using directional ligations or the QuikChange Site-Directed Mutagenesis Kit (Stratagene, La Jolla, CA). Oligonucleotide primers containing a mutation were designed with a change in a silent restriction site to allow rapid identification of the mutant. Incorporation of the mutation into the template was then confirmed by DNA sequencing. Double mutants were made by subcloning the region of DIV-S6 that contained the mutation into the rNa_V_1.4-K1237E construct. All constructs were linearized by either *Spe*I or *Sal*I and transcribed with either T7 (*Spe*I-linearized) or SP6 (*Sal*I-linearized) RNA Polymerase using the respective mMessage Machine Kits according to the manufacturer’s protocols (Ambion, Austin, TX).

### Two-microelectrode voltage clamp recording in *Xenopus* oocytes

Two-microelectrode voltage-clamp was performed in all experiments except those presented in Figs 3 and 5, in which the patch clamp method was used in order to provide a better quality of the clamp. The use of the two-microelectroce technique was necessary because the constructs containing the mutation K1237E only poorly expressed in mammalian cells. Stage V and VI *Xenopus laevis oocytes* were isolated from female frogs (NASCO, Ft. Atkinson, WI), washed with Ca^2+^-free solution (90 mM NaCl, 2.5 mM KCl, 1 mM MgCl_2_, 1 mM NaHPO_4_, and 5 mM HEPES titrated to pH 7.6 with 1 N NaOH), treated with 2 mg/ml collagenase (Sigma, St. Louis, MO) for 1.5 h, and had their follicular cell layers manually removed. As judged from photometric measurements, approximately 50–100 ng of native or mutant α subunit cRNA was injected into each oocyte with a Drummond micro-injector (Broomall, PA). Oocytes were incubated at 17 °C for 12 h to 14 days before examination.

Recordings were made in the two-electrode voltage clamp configuration using a TEC 10CD clamp (npi electronic, Tamm, Germany). For accurate adjustment of the experimental temperature (18 ± 0.5 °C) an oocyte bath cooling system (HE 204, Dagan, Minneapolis, MN) was used. Oocytes were placed in recording chambers in which the bath flow rate was about 100 ml/h, and the bath level was adjusted so that the total bath volume was less than 500 µl. Electrodes were filled with 3 M KCl and had resistances of less than 0.5 MΩ. Using pCLAMP6 (Axon Instruments, Foster City, CA) software, data were acquired at 71.4 kHz after low-pass filtration at 2 kHz (- 3dB). Recordings were made in a bathing solution that consisted of (in mM): 90 NaCl, 2.5 KCl, 1 BaCl_2_, 1 MgCl_2_ and 5 mM HEPES titrated to pH 7.2 with 1 N NaOH. BaCl_2_ was used as a replacement for CaCl_2_ in order to minimize Ca^2+^-activated Cl^−^ currents.

### Whole-cell patch-clamp recording

TsA201 cells were grown in Dulbecco’s modified Eagle’s medium supplemented with 10% fetal bovine serum and 20 units/ml each of penicillin and streptomycin (Gibco, Gaithersburg, Md., USA). Cells were maintained at 37 °C in a humid atmosphere containing 5% CO2. Prior to recording, cells were dissociated from their substrate by treatment with a 0.25% trypsin solution (Gibco) for approximately 2 min, pelleted, resuspended in bath solution, and allowed to settle to the bottom of the recording chamber. Channel DNA was transiently transfected into tsA201 cells using ExGene 500 *in vitro* transfection reagent (Fermentas, Thermo Scientific).

Na^+^ currents were recorded using an Axopatch 200B patch clamp amplifier (Axon Instruments, Union City, CA) as in^67^. Recording was begun about 10 minutes after whole cell access was attained in order to minimize time-dependent shifts in gating. Pipettes were formed from aluminosilicate glass (AF150-100-10; Science Products, Hofheim, Germany) with a P-97 horizontal puller (Sutter Instruments, Novato, CA), heat-polished on a microforge (MF-830; Narishige, Japan), and had resistances between 1 and 2 MΩ when filled with the recording pipette solution (105 mM CsF, 10 mM NaCl, 10 mM EGTA, 10 mM HEPES, pH = 7.3). Peak current amplitudes equaled −1.2 ± 0.4 nA. Series resistance was minimized ( > 80–90%) using the Axoclamp 200B device. As such the uncompensated voltage error across the pipette was calculated to be 3.2 ± 0.3 mV. The bath solution consisted of (mM): 140 NaCl, 2.5 KCl, 1 CaCl_2_, 1 MgCl_2_ 10 HEPES. Voltage-clamp protocols and data acquisition were performed with pclamp 10.0 software (Axon Instruments, Molecular Devices) through a 12-bit A-D/D-A interface (Digidata 1200; Axon Instruments). Data were low-pass filtered at 2 kHz (−3 dB) and digitized at 10–20 kHz.

### Electrophysiologic protocols

The holding potential was −120 mV and −140 mV with two electrode voltage clamp in *Xenopus laevis* oocytes and patch-clamp in mammalian cells, respectively.

The voltage-dependence of steady-state fast inactivation was assessed by application of 50 ms conditioning pulses to varying potentials followed by a 10 ms test pulse to −20 mV. The test pulse duration of 50 ms has previously been shown to drive channels into fast inactivation with minimal contribution of slow inactivation^39^.

The time course of recovery from inactivation was assessed by application of a conditioning pulse to −20 mV followed after a varying interval at the holding potential of −120 mV by 10 ms test pulses to −20 mV. The duration of the conditioning prepulse was 100 ms and 1 s for the assessment of recovery from faster and slower inactivated states, respectively. For determination of the voltage-dependence of activation cells were depolarized for 20 ms to potentials between −100 mV and +70 mV at 10 mV increments.

### Data evaluation

For data evaluation the normalized peak currents were subjected to the following analyses:

Steady-state inactivation data were fitted with a Boltzmann function:

1 $${\rm{y}}=1/(1+\exp (-\text{zF}/\text{RT}({\rm{V}}-{{\rm{V}}}_{1/2})))$$

where V is the voltage of the conditioning prepulse, V_1/2_ is the voltage at which half-maximum inactivation occurred, *z* is the effective valence, F is Faraday´s constant, R is the gas constant, and T is room temperature in °K.

Recovery from fast and slow inactivation:

2 $${\rm{y}}=-{{\rm{A}}}_{1}(1-\exp (-{\rm{t}}/{\tau }_{1}))-{{\rm{A}}}_{2}(1-\exp (-{\rm{t}}/{\tau }_{2}))+{\rm{C}}$$

3 $${\rm{y}}=-{{\rm{A}}}_{1}(1-\exp (-{\rm{t}}/{\tau }_{1}))-{{\rm{A}}}_{2}(1-\exp (-{\rm{t}}/{\tau }_{2})\,\mbox{--}\,{{\rm{A}}}_{3}(1-\exp (-t/{\tau }_{3}))+{\rm{C}}$$

where τ_1_, τ_2_ and τ_3_ are the time constants of distinct components of recovery, A_1_, A_2_ and A_3_ are the respective amplitudes of these components and C is the final level of recovery.

Current-voltage relationships were fit with the function,

4 $${{\rm{G}}}_{{\rm{\max }}}\ast ({\rm{V}}-{{\rm{V}}}_{{\rm{rev}}})\ast (1-(1/(1+\exp (({\rm{V}}-{{\rm{Va}}}_{1/2})/{\rm{K}})))),$$

where V is the step potential, G_max_ is the maximum conductance, Va_1/2_ is the voltage at which half-maximum activation occurred, V_rev_ is the reversal potential, and K is the slope factor.

Curve fitting was performed using ORIGIN 7.5 (MicroCal Software, Inc., Northampton, MA).

### Mutant cycle analysis

V_1/2_ and *z* values from Eq. 3 were used to calculate ΔG for inactivation:

5 $${\rm{\Delta }}{\rm{G}}={{\rm{zFV}}}_{1/2}$$

the standard error of ΔG, SE_ΔG_, is given by

6 $${{\rm{SE}}}_{{\rm{\Delta }}G}={{\rm{\Delta }}{\rm{G}}((\text{SE}}_{{\rm{z}}}/{\rm{z}}{)}^{2}+{({{\rm{SE}}}_{{\rm{V}}1/2}{/{\rm{V}}}_{1/2})}^{2}{)}^{0.5}$$

where SE refers to standard error

The free energy of coupling (ΔΔG_coupling_) between mutantans in DIV-S6 (MUT1) and K1237E (MUT2) is given by

7 $${{\rm{\Delta }}{\rm{\Delta }}{\rm{G}}}_{{\rm{coupling}}}=({{\rm{\Delta }}{\rm{G}}}_{{\rm{MUT}}1}-{{\rm{\Delta }}{\rm{G}}}_{{\rm{WT}}})\mbox{--}({{\rm{\Delta }}{\rm{G}}}_{{\rm{MUT}}1/{\rm{MUT}}2}-{{\rm{\Delta }}{\rm{G}}}_{/{\rm{MUT}}2})$$

and the associated standard error of ΔΔG_coupling_ is given by

8 $${{\rm{SE}}}_{{\rm{\Delta }}{\rm{\Delta }}{\rm{G}}-\text{coupling}}={({{{\rm{SE}}}_{{\rm{\Delta }}{\rm{G}},\text{MUT}1}}^{2}+{{{\rm{SE}}}_{{\rm{\Delta }}\text{GMUT}2}}^{2})}^{0.5}$$

### Statistics

Data are expressed as means ± S.E.M. Statistical comparisons were made using the two-tailed Student’s t-tests. A P < 0.05 was considered as being significant.

### Molecular Modeling

The coordinates of the inactivated NavAb crystal structure in conformation AB (PDB Entry: 4EKW, Resolution: 3.2 Å) was used as starting point to generate a model of the rat Nav1.4 channel^29^. One of the chains where the highly conserved asparagine 211 (N1584 in Nav1.4) forms a hydrogen bond with the S4-S5 linker from the adjacent subunit was replaced with rat Nav1.4 sequence.

Molecular dynamics (MD) simulations were performed with Gromacs version 4.5.4^68^ and all charged residues were treated keeping their charge states at physiological pH 7.4. The valine mutant at position 1581 was generated with Pymol^69^. The models were subjected to 3 × 100 ns MD simulations with 250 mM NaCl solution. Simulations were carried out with the AMBER99sb^70^ all atom force field. 1-palmitoyl-2-oleoylphosphatidylcholine (POPC) lipids parameters were derived from Berger^71,72^ and the TIP3P water model was applied^73^.

All covalent bonds were constrained using the LINCS algorithm^74^, allowing for an integration time step of 2 fs. A 10 Å cutoff was adopted for calculating short-range electrostatic interactions and the Particle Mesh Ewald^75^ summation was used for calculating long-range electrostatic interactions. The corrected Lennard-Jones ion parameters for the amber forcefield^76^ were implemented in this study and the vdW interactions were calculated with a cutoff of 10 Å. The Nose-Hoover thermostat^77,78^ and the semi-isotropic Parrinello-Rahman barostat algorithm^79^ was used to maintain simulation temperature and pressure constantly at 310 K and 1 bar, respectively. Prior to MD simulations, 3000 conjugate gradient energy-minimization steps were performed, followed by 5 ns equilibration in order to fully solvate mobile water and lipids around a restrained protein with a force constant of 1000 kJ/mol/nm^2^ on all heavy atoms.

### Data availability

The datasets generated during and/or analysed during the current study are available from the corresponding author upon resonable request.

### Ethical approval

This study did not use any live vertebrates and the methods used in this study were in accordance with the respective guidelines and regulations of the University Animal Welfare Committee of Medical University of Vienna.

## Electronic supplementary material

Supplementary Figures

## References

[1] West JW (1992). A cluster of hydrophobic amino acid residues required for fast Na^+^-channel inactivation. Proc Natl Acad Sci USA.

[2] Kellenberger S, Scheuer T, Catterall WA (1996). Movement of the Na^+^ channel inactivation gate during inactivation. J Biol Chem.

[3] Cannon SC, Strittmatter SM (1993). Functional expression of sodium channel mutations identified in families with periodic paralysis. Neuron.

[4] McPhee JC, Ragsdale DS, Scheuer T, Catterall WA (1994). A mutation in segment IVS6 disrupts fast inactivation of sodium channels. Proc Natl Acad Sci USA.

[5] McPhee JC, Ragsdale DS, Scheuer T, Catterall WA (1995). A critical role for transmembrane segment IVS6 of the sodium channel alpha subunit in fast inactivation. J Biol Chem.

[6] Hayward LJ, Brown RH, Cannon SC (1997). Slow inactivation differs among mutant Na channels associated with myotonia and periodic paralysis. Biophys J.

[7] Wright SN, Wang SY, Wang GK (1998). Lysine point mutations in Na^+^ channel D4-S6 reduce inactivated channel block by local anesthetics. Mol Pharmacol.

[8] Vedantham V, Cannon SC (2000). Rapid and slow voltage-dependent conformational changes in segment IVS6 of voltage-gated Na^+^ channels. Biophys J.

[9] Todt H, Dudley SC, Kyle JW, French RJ, Fozzard HA (1999). Ultra-slow inactivation in µ1 Na^+^ channels is produced by a structural rearrangement of the outer vestibule. Biophys J.

[10] Zarrabi T (2010). A molecular switch between the outer and the inner vestibules of the voltage-gated Na^+^ channel. J Biol Chem.

[11] Heinemann SH, Terlau H, Stühmer W, Imoto K, Numa S (1992). Calcium channel characteristics conferred on the sodium channel by single mutations. Nature.

[12] Favre I, Moczydlowski E, Schild L (1996). On the structural basis for ionic selectivity among Na^+^, K^+^, and Ca^2+^ in the voltage-gated sodium channel. Biophys J.

[13] Schlief T, Schoenherr R, Imoto K, Heinemann SH (1996). Pore properties of rat brain II sodium channels mutated in the selectivity filter domain. Eur Biophys J.

[14] Chen S, Hartmann HA, Kirsch GE (1997). Cysteine mapping in the ion selectivity and toxin binding region of the cardiac Na^+^ channel pore. J Membr Biol.

[15] Tsushima RG, Li RA, Backx PH (1997). Altered ionic selectivity of the sodium channel revealed by cysteine mutations within the pore. J Gen Physiol.

[16] Szendroedi J (2007). Speeding the recovery from ultraslow inactivation of voltage-gated Na^+^ channels by metal ion binding to the selectivity filter: a foot-on-the-door?. Biophys J.

[17] Hilber K (2005). *S*electivity filter residues contribute unequally to pore stabilization in voltage-gated sodium channels. Biochemistry.

[18] Nuss HB (1996). Coupling between fast and slow inactivation revealed by analysis of a point mutation (F1304Q) in μ1 rat skeletal muscle sodium channels. J Physiol.

[19] Armstrong CM, Bezanilla F (1977). Inactivation of the sodium channel. II. Gating current experiments. J Gen Physiol.

[20] Popa MO, Alekov AK, Bail S, Lehmann-Horn F, Lerche H (2004). Cooperative effect of S4-S5 loops in domains D3 and D4 on fast inactivation of the Na^+^ channel. J Physiol.

[21] Piper DR, Rupp J, Sachse FB, Sanguinetti MC, Tristani-Firouzi M (2008). Cooperative interactions between R531 and acidic residues in the voltage sensing module of hERG1 channels. Cell Physiol Biochem.

[22] Smith MR, Goldin AL (1997). Interaction between the sodium channel inactivation linker and domain III S4–S5. Biophys J.

[23] Gebauer M (2004). *N*-type inactivation features of Kv4.2 channel gating. Biophys J.

[24] Hidalgo P, MacKinnon R (1995). Revealing the architecture of a K^+^ channel pore through mutant cycles with a peptide inhibitor. Science.

[25] Yifrach O, MacKinnon R (2002). Energetics of pore opening in a voltage-gated K^+^ channel. Cell.

[26] Starace DM, Stefani E, Bezanilla F (1997). Voltage-dependent proton transport by the voltage sensor of the Shaker K^+^ channel. Neuron.

[27] Vilin YY, Peters CH, Ruben PC (2012). Acidosis differentially modulates inactivation in Nav1.2, Nav1.4, and Nav1.5 channels. Front Pharmacol.

[28] Payandeh J, Scheuer T, Zheng N, Catterall WA (2011). The crystal structure of a voltage-gated sodium channel. Nature.

[29] Payandeh J, Gamal El-Din TM, Scheuer T, Zheng N, Catterall WA (2012). Crystal structure of a voltage-gated sodium channel in two potentially inactivated states. Nature.

[30] McCusker EC (2012). *S*tructure of a bacterial voltage-gated sodium channel pore reveals mechanisms of opening and closing. Nat Commun.

[31] Sula A (2017). *T*he complete structure of an activated open sodium channel. Nat Commun.

[32] Lenaeus MJ (2017). *S*tructures of closed and open states of a voltage-gated sodium channel. Proc Natl Acad Sci USA.

[33] Shen H. *et al*. *S*tructure of a eukaryotic voltage-gated sodium channel at near-atomic resolution. *Science* **355** (2017). 10.1126/science.aal4326 28183995

[34] Yan Z (2017). *S*tructure of the Nav1.4-beta1 complex from electric eel. Cell.

[35] Armstrong CM (2006). Na channel inactivation from open and closed states. Proc Natl Acad Sci USA.

[36] Sula A, Wallace BA (2017). Interpreting the functional role of a novel interaction motif in prokaryotic sodium channels. J Gen Physiol.

[37] O’Leary ME (1998). Characterization of the isoform-specific differences in the gating of neuronal and muscle sodium channels. Can J Physiol Pharmacol.

[38] Yarov-Yarovoy V (2002). Role of amino acid residues in transmembrane segments IS6 and IIS6 of the Na^+^ channel alpha subunit in voltage-dependent gating and drug block. J Biol Chem.

[39] Wang SY, Bonner K, Russell C, Wang GK (2003). Tryptophan scanning of D1S6 and D4S6 C-termini in voltage-gated sodium channels. Biophys J.

[40] Goldschen-Ohm MP, Capes DL, Oelstrom KM, Chanda B (2013). Multiple pore conformations driven by asynchronous movements of voltage sensors in a eukaryotic sodium channel. Nat Commun.

[41] Chen LQ, Santarelli V, Horn R, Kallen RG (1996). A unique role for the S4 segment of domain 4 in the inactivation of sodium channels. J Gen Physiol.

[42] Chahine M (1994). *S*odium channel mutations in paramyotonia congenita uncouple inactivation from activation. Neuron.

[43] Chanda B, Bezanilla F (2002). Tracking voltage-dependent conformational changes in skeletal muscle sodium channel during activation. J Gen Physiol.

[44] Sheets MF, Kyle JW, Kallen RG, Hanck DA (1999). The Na^+^ channel voltage sensor associated with inactivation is localized to the external charged residues of domain IV, S4. Biophys J.

[45] McPhee JC, Ragsdale DS, Scheuer T, Catterall WA (1998). A critical role for the S4–S5 intracellular loop in domain IV of the sodium channel alpha-subunit in fast inactivation. J Biol Chem.

[46] Lerche H (1997). Role in fast inactivation of the IV/S4-S5 loop of the human muscle Na^+^ channel probed by cysteine mutagenesis. J Physiol.

[47] Bosmans F, Martin-Eauclaire MF, Swartz KJ (2008). Deconstructing voltage sensor function and pharmacology in sodium channels. Nature.

[48] Sunami A, Dudley SC, Fozzard HA (1997). Sodium channel selectivity filter regulates antiarrhythmic drug binding. Proc Natl Acad Sci USA.

[49] Tsang SY, Tsushima RG, Tomaselli GF, Li RA, Backx PH (2005). A multifunctional aromatic residue in the external pore vestibule of Na^+^ channels contributes to the local anesthetic receptor. Mol Pharmacol.

[50] Benitah JP, Chen Z, Balser JR, Tomaselli GF, Marban E (1999). Molecular dynamics of the sodium channel pore vary with gating: interactions between P-segment motions and inactivation. J Neurosci.

[51] Lukacs P (2014). Exploring the Structure of the Voltage-gated Na^+^ Channel by an Engineered Drug Access Pathway to the Receptor Site for Local Anesthetics. J Biol Chem.

[52] Zhang X (2012). *C*rystal structure of an orthologue of the NaChBac voltage-gated sodium channel. Nature.

[53] French RJ (1996). *I*nteractions between a pore-blocking peptide and the voltage sensor of the sodium channel: an electrostatic approach to channel geometry. Neuron.

[54] Khan A, Romantseva L, Lam A, Lipkind G, Fozzard HA (2002). Role of outer ring carboxylates of the rat skeletal muscle sodium channel pore in proton block. J Physiol.

[55] Oelstrom K, Goldschen-Ohm MP, Holmgren M, Chanda B (2014). Evolutionarily conserved intracellular gate of voltage-dependent sodium channels. Nat Commun.

[56] O’Leary ME, Chahine M (2015). MTSET modification of D4S6 cysteines stabilize the fast inactivated state of Nav1.5 sodium channels. Front Pharmacol.

[57] Cha A, Ruben PC, George ALJ, Fujimoto E, Bezanilla F (1999). Voltage sensors in domains III and IV, but not I and II, are immobilized by Na^+^ channel fast inactivation. Neuron.

[58] Capes DL, Goldschen-Ohm MP, rcisio-Miranda M, Bezanilla F, Chanda B (2013). Domain IV voltage-sensor movement is both sufficient and rate limiting for fast inactivation in sodium channels. J Gen Physiol.

[59] Horn R, Ding S, Gruber HJ (2000). Immobilizing the moving parts of voltage-gated ion channels. J Gen Physiol.

[60] Yang N, Horn R (1995). Evidence for voltage-dependent S4 movement in sodium channels. Neuron.

[61] Catterall WA, Goldin AL, Waxman SG (2005). International Union of Pharmacology. XLVII. Nomenclature and structure-function relationships of voltage-gated sodium channels. Pharmacol Rev.

[62] Creighton, T. E. Proteins. Structures and Molecular Properties. New York: W. H. Freeman and Company, 1993.

[63] Hsu EJ (2017). Regulation of Na^+^ channel inactivation by the DIII and DIV voltage-sensing domains. J Gen Physiol.

[64] Chang CC (2004). *A* novel SCN5A mutation manifests as a malignant form of long QT syndrome with perinatal onset of tachycardia/bradycardia. Cardiovascular Research.

[65] Misra SN, Kahlig KM, George AL (2008). Impaired NaV1.2 function and reduced cell surface expression in benign familial neonatal-infantile seizures. Epilepsia.

[66] Scalmani P (2006). Effects in neocortical neurons of mutations of the Nav1.2 Na^+^ channel causing benign familial neonatal-infantile seizures. J Neurosci.

[67] Zebedin, E. *et al*. Fiber type conversion alters inactivation of voltage-dependent sodium currents in mouse C2C12 skeletal muscle cells. *Am J Physiol Cell Physiol* (2004). 10.1152/ajpcell.00015.2004 15044148

[68] Hess B, Kutzner C, Van Der Spoel D, Lindahl E (2008). GROMACS 4: Algorithms for highly efficient, load-balanced, and scalable molecular simulation. J Chem Theory Comput.

[69] Schroedinger, L. L. C. The PyMOL molecular graphics system, version 1.3r1. Schroedinger, LLC, Portland, OR. 2010.

[70] Hornak V (2006). *C*omparison of multiple Amber force fields and development of improved protein backbone parameters 69. Proteins.

[71] Cordomi A, Caltabiano G, Pardo L (2012). Membrane protein simulations using AMBER force field and Berger lipid parameters. J Chem Theory Comput.

[72] Bachar M, Brunelle P, Tieleman DP, Rauk A (2004). Molecular Dynamics Simulation of a Polyunsaturated Lipid Bilayer Susceptible to Lipid Peroxidation. J Phys Chem B.

[73] Jorgensen WL, Chandrasekhar J, Madura JD, Impey RW, Klein ML (1983). Comparison of simple potential functions for simulating liquid water. J Chem Phys.

[74] Hess B, Bekker H, Berendsen HJC, Fraaije JGEM (1997). LINCS: A linear constraint solver for molecular simulations. J Comput Chem.

[75] Darden T, York D, Pedersen L (1993). Particle mesh Ewald: An N log (N) method for Ewald sums in large systems. J Chem Phys.

[76] Joung IS, Cheatham TE (2008). Determination of alkali and halide monovalent ion parameters for use in explicitly solvated biomolecular simulations. J Phys Chem.

[77] Nose S, Nose S (1984). A unified formulation of the constant temperature molecular-dynamics methods. J Chem Phys.

[78] Hoover WG, Hoover WG (1985). Canonical dynamics: Equilibrium phase-space distributions. Phys Rev.

[79] Martonak R, Laio A, Parrinello M (2003). Predicting crystal structures: the Parrinello-Rahman method revisited. Phys Rev Lett.

