# Supplementary material for: Distinct modulation of inactivation by a residue in the pore domain of voltage-gated Na+ channels: mechanistic insights from recent crystal structures
Source: Sci Rep. 2018 Jan 12;8:631. doi: 10.1038/s41598-017-18919-1 (PMC5766632; doi:10.1038/s41598-017-18919-1)

**Distinct modulation of inactivation by a residue in the pore domain of voltage-gated Na^+^channels: mechanistic insights from recent crystal structures**

**Rene Cervenka, Peter Lukacs, Vaibhavkumar S. Gawali, Song Ke, Xaver Koenig, Lena Rubi, Touran Zarrabi, Karlheinz Hilber, Walter Sandtner, Anna Stary-Weinzinger, Hannes Todt**

**SUPPLEMENTARY FIGURES**

**Figure legends for supplemental figues.**

Supplementary Figure 1

Alignment of voltage-gated Na^+^ sequences of DIV-S6 of three mammalian species. The shaded area denotes a stretch of amino acid with 100% sequence homology across isoforms and species with the exception of I1581 in rNav 1.4, which is replaced by valines in several isoforms (yellow shading, bold)

Supplementary Figure 2

Molecular dynamics simulations suggest that the mutation I1581V are may result in an altered interaction between site 1581 and L1146. Non-bonded Interaction energies of residues within 3.5 Å of site 1581 were calculated using 100ns trajectories (25ps interval) from molecular dynamics simulations in a NavAb-based homology model. The greatest relative change in interaction energies by I1581V and residues in the DIII S4-S5 linker is observed with L1146 which is in good accordance with the structural data shown in Fig. 7.

Figure S1


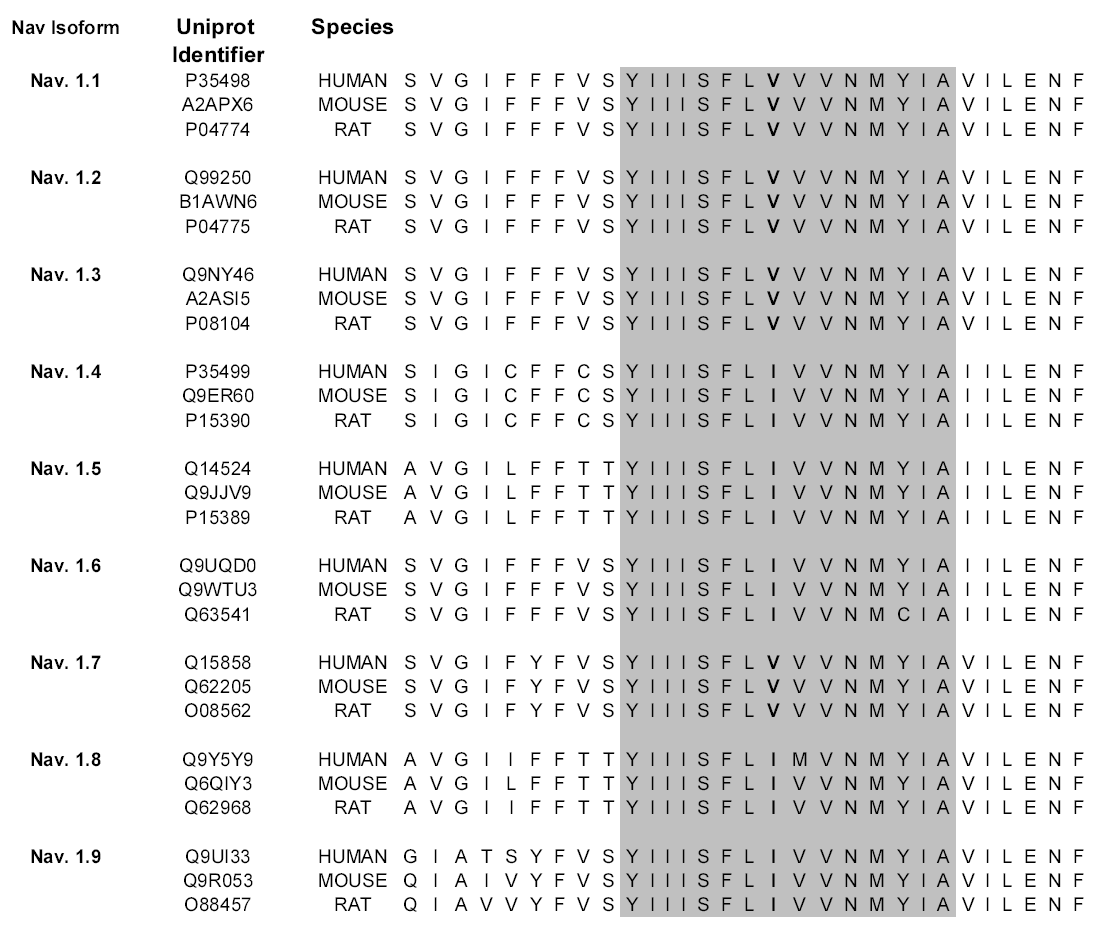


Figure S2


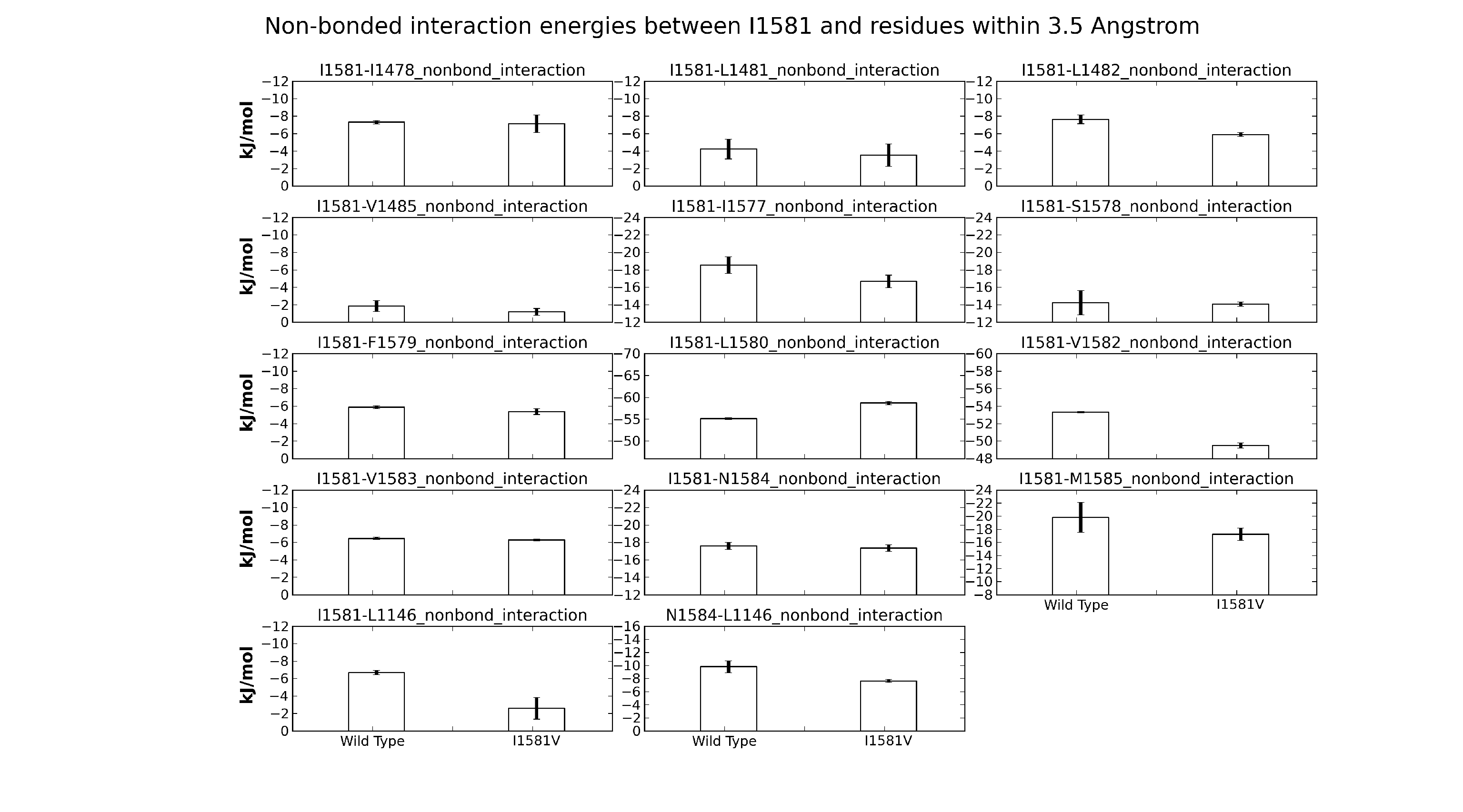

Supplement: Supplementary file 1 — Supplementary Figures [file 41598_2017_18919_MOESM1_ESM.doc]
